# A Sensing Element Based on a Bent and Elongated Grooved Polymer Optical Fiber

**DOI:** 10.3390/s120607485

**Published:** 2012-06-01

**Authors:** Wei-Hua Lu, Li-Wen Chen, Wen-Fu Xie, Yung-Chuan Chen

**Affiliations:** 1 Department of Material Engineering, National Pingtung University of Science and Technology, Pingtung 91201, Taiwan; E-Mail: whl@mail.npust.edu.tw; 2 Department of Vehicle Engineering, National Pingtung University of Science and Technology, Pingtung 91201, Taiwan; E-Mails: liwen@mail.npust.edu.tw (L.-W.C.); m9538026@alumni.npust.edu.tw (W.-F.X.)

**Keywords:** plastic optical fiber, bending and elongation, displacement sensor

## Abstract

An experimental and numerical investigation is performed into the power loss induced in grooved polymer optical fibers (POFs) subjected to combined bending and elongation deformations. The power loss is examined as a function of both the groove depth and the bend radius. An elastic-plastic three-dimensional finite element model is constructed to simulate the deformation in the grooved region of the deformed specimens. The results indicate that the power loss increases significantly with an increasing bending displacement or groove depth. Specifically, the power loss increases to as much as 12% given a groove depth of 1.1 mm and a bending displacement of 10 mm. Based on the experimental results, an empirical expression is formulated to relate the power loss with the bending displacement for a given groove depth. It is shown that the difference between the estimated power loss and the actual power loss is less than 2%.

## 1. Introduction

Compared to conventional glass fibers, polymer optical fibers (POFs) have a larger core diameter, greater flexibility, lighter weight, and a lower cost [1], and as a result, POFs have attracted intensive interest for sensing applications in recent years [2–18]. Arrue *et al.* [2] showed that the power loss induced in bent POFs increases as the bend radius is reduced. Zubia *et al.* [3] proposed a POF-based barrier sensor for detecting the wind speed in a wind generator. Lomer *et al.* [4] developed a quasi-distributed system for level sensing based on a bent side-polished POF cable. Kuang *et al.* [5] presented a POF sensor for crack detection and vertical deflection monitoring in concrete beams under flexural loading conditions. Kulkarni *et al.* [6] developed a novel POF sensor for weight measurement applications and showed that the sensitivity could be enhanced by increasing the corrugation pitch of the deforming plates used within the sensor structure. Babchenko and Maryles [7] presented a displacement sensor based on a bent imperfected POF. The experimental results showed that the sensitivity of the device was critically dependent on the abrasion angle, location angle, and displacement of the 3D imperfections. Losada *et al.* [8] showed that the application of strain to a POF causes a reduction in the bandwidth and an increase in the power loss. Kuang *et al.* [9] developed a POF displacement sensor based on dual cycling bending and showed that the sensitivity could be enhanced by increasing the number of rollers or decreasing the interval between the rollers. Daum *et al.* [10] reported that the power loss in POFs subject to tensile loading reduces by no more than 2∼3% prior to the onset of plastic deformation. Chen *et al.* [11] showed that the sensitivity of POFs subjected to cyclic tensile loading increases with an increasing load or a greater number of cycles. Cennamo *et al.* [12] developed a POF sensor as a biosensor. This sensor was implemented based on Surface Plasmon Resonance (SPR) at the interface between a liquid sample and a thin gold layer deposited on the core of a POF. The result indicated that the sensitivity of the optical sensor depended on the length of the sensing region as well as the thickness of the gold film. Zhu *et al.* [13] showed a POF displacement sensor based on cycling bending for landslides monitoring with a bowknot at one POF end. The results showed that the sensitivity of the sensor increased as the dimension of the bowknot decreased. Montero *et al.* [14] presented a novel POF self-referencing fiber optic intensity sensor based on bending losses of a partially polished POF coupler for liquid detection. The results showed that this technique increased the external media refractive index sensitivity of the sensor. Vijayan *et al.* [15] demonstrated the potential of using the POF macrobend for measuring weight. It was found that though elongative bending decreased the output intensity, compressive bending gave a reverse effect.

In all of the above studies, the sensitivity of the proposed POF sensors was enhanced by making the POFs imperfect in some way. In previous studies [16–18], the present group performed experimental and numerical investigations into the power loss characteristics of bent and elongated POFs with no grooves. The results showed that the power loss increases significantly as the bend radius is reduced or the elongation increased. However, the literature contains little information regarding the sensitivity of grooved POFs under combined bending and elongation conditions. Accordingly, the present study performs a series of experimental tests to evaluate the power loss induced in bent and elongated POFs with groove depths ranging from 0 mm to 1.1 mm. The deformation which takes place near the grooved region of the various specimens is analyzed using a three-dimensional finite element (FE) model. In addition, the experimental results are used to construct an empirical model for relating the power loss to the bend displacement given a known groove depth. The validity of the proposed model is confirmed by comparing the estimated value of the power loss with the measured value for various values of the groove depth and bend displacement.

## 2. Experimental Setup

**Figure 1.**
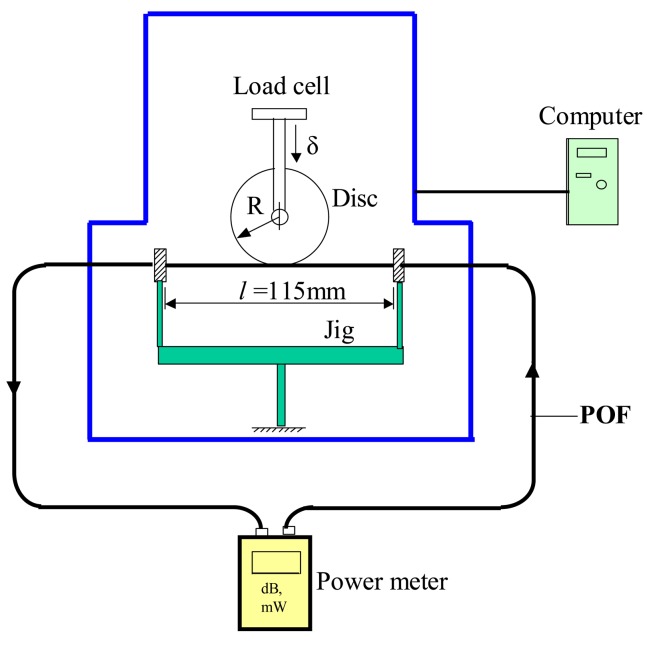
Experimental setup used to measure power loss in grooved POF specimens under combined bending and elongation loading.

Figure 1 presents a schematic illustration of the experimental setup used to measure the power loss in the bent and elongated grooved POF specimens. The major items of equipment include a tensile test machine (EZ Test-500N, Shimadzu, Kyoto, Japan), a disc, a computer system and an optical power meter (Photom, model 205A, Tokyo, Japan). The elongation tests were performed using four discs with different radii, namely R = 5, 10, 15 and 20 mm. The POF specimens (step index type, SH-4001, Mitsubishi Rayon Company Ltd.) had a coating diameter of 2.2 mm, a cladding diameter of 1 mm, a core diameter of 0.98 mm, and a numerical aperture (NA) of 0.5. The core, cladding and coating of these POFs were fabricated from polymethyl methacrylate (PMMA), polytetrafluoroethylene (PTFE) and low-density polyethylene (LDPE), respectively. The refractive indices of core and cladding are *n_co_* = 1.492 and *n_cl_* = 1.402, respectively. Each POF specimen had a total length of 800 mm. Prior to the elongation tests, the POF specimens were clamped in such a way as to create a gauge length of 115 mm. One of the ends of the specimen was then connected to the light source (a light emitting diode with a wavelength of 660 nm), while the other was connected to a power detector. In each test, the center of the disc was carefully aligned with the center of the POF gauge length and the disc was then displaced through a distance of 10 mm in the vertical (downward) direction.

**Figure 2.**
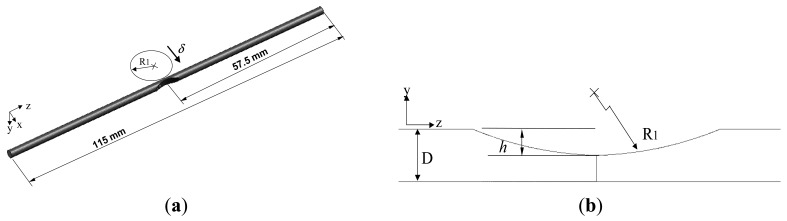
Geometrical model of grooved POF specimen. (**a**) Gauge length of POF specimen with grooved section located at mid-point position. (**b**) Enlarged view of groove geometry.

The groove-like features in the POF specimens were produced using a grinding wheel (diamond grain size: #120) and therefore had a curved profile. Figure 2 presents a geometrical model of a typical grooved specimen. As shown in Figure 2(a), the groove was formed at the mid-point position of the gauge length. Figure 2(b) presents an enlarged view of the groove geometry, in which *R_1_* is the radius of curvature of the groove, *D* is the external diameter of the POF, and *h* is the depth of the groove as measured from the top surface of the POF. In the present study, *D* = 2.2 mm, *R_1_* = 7.5 mm, and the groove depth was assigned values of *h* = 0, 0.7, 0.9 and 1.1 mm. The variations in attenuation and disc displacement are recorded synchronically by the power meter and the PC, respectively.

## 3. Finite Element Model

**Figure 3.**
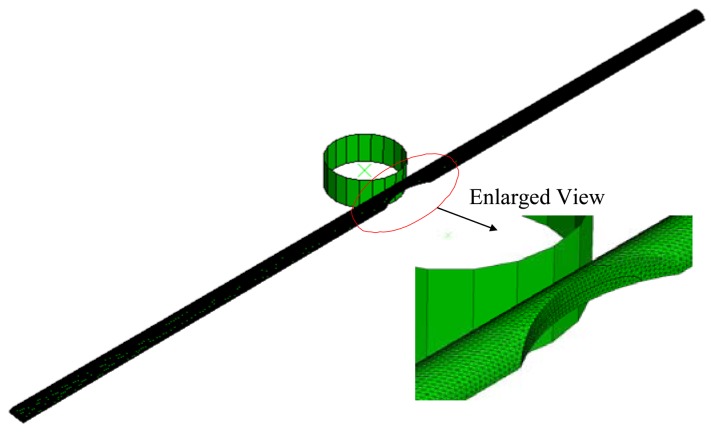
Finite element model of grooved POF specimen.

In this study, an elastic-plastic finite element model is employed to evaluate the deformation and stress distributions in the core diameter as the POF specimen is subjected to bent and elongated deformation. The simulations are performed using the commercial finite element package ABAQUS. The finite element mesh of the tested POF specimen is shown in Figure 3.

The disc is modeled as an analytical surface rigid body and the fiber model is constructed using four-node, three-dimensional tetrahedron elements. In performing the simulations, the contact behavior between the disc and the POF is modeled using surface-to-surface contact. Due to the symmetry of the POF geometry, only one half of the model needs be considered in the finite element analysis. Various finite element mesh sizes are performed for the convergence test of the von-Mises stress computed at the center point of the grooved POF specimen. The exact number of elements used in the simulations depends on the depth of groove. However, in general, the simulations involve approximately 246,484 elements and 61,706 nodes. In the finite element analysis, both ends of the specimen is considered to be fixed, while a displacement of *δ* is applied in the x direction to the center of the disc. The mechanical properties of the core, cladding and coating used in the current finite element simulations are summarized in Table 1.

## 4. Results and Discussions

In the experimental tests, POF specimens with groove depths of *h* = 0, 0.7, 0.9 and 1.1 mm, respectively, were bent and elongated through a total displacement of *δ* = 10 mm using discs with radii of *R* = 5, 10, 15 and 20 mm, respectively. The power delivered to the POF specimen with no groove prior to elongation was measured in advance and denoted as *P_in_* (33 *μW*). The variation in measured power value *P_in_* for the used light source is about 33 *μW* ± 0.2 *μW*. The output power *P_out_* was then measured continuously as the fiber was elongated. The relative humidity (RH) at temperatures of 25 °C is 61% in experiments. Although the *P_in_* value is affected by the RH, according to our experiment results, the variation of power ratio *P_out_*/*P_in_* with bend and elongation is independent of the RH. Meanwhile, the fibers suffer lower power losses at higher temperatures from experiment results. According to our experiment results, the variation of power ratio is nearly independent of the temperature as the ambient temperature is below 40 °C.

The initial power ratio (*P_out_*/*P_in_*) at *δ* = 0 mm was calculated and denoted as *η_o_* = (*P_out_*/*P_in_*)*_δ=0_*. The initial power ratios of the POF specimens with groove depths of *h* = 0.0, 0.7, 0.9 and 1.1 mm were found to be (*P_out_*/*P_in_*)*_δ=0_* = 1.00, 0.92, 0.80, and 0.56, respectively. To compare the relationship between the power loss and the bending elongation in the different grooved POF specimens, the power ratio *η* = *P_out_*/*P_in_* was normalized as follows:

(1) $$\bar{\eta }=\frac{\eta }{\eta_{o}}$$

**Figure 4.**
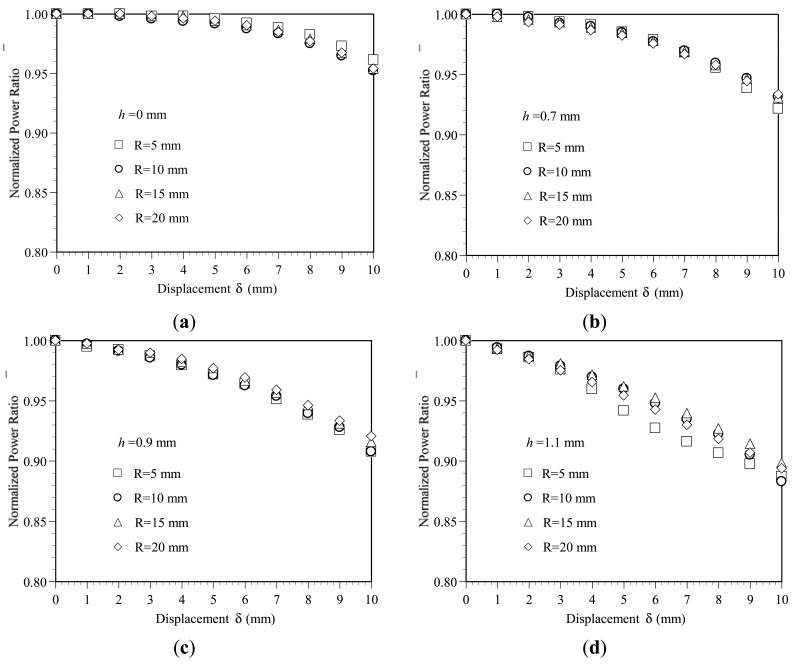
Variation of normalized power ratio *η̄* with displacement *δ* as function of disc radius *R* given groove depths of: (**a**) *h* = 0 mm. (**b**) *h* = 0.7 mm. (**c**) *h* = 0.9 mm. (**d**) *h* = 1.1 mm.

Figure 4 plots the variation of the normalized power ratio *η̄* with the bending displacement *δ* as a function of the bend radius *R* given groove depths of *h* = 0, 0.7, 0.9 and 1.1 mm, respectively. It is seen that for all values of the groove depth, the power loss increases with an increasing bending displacement. The power loss is particularly significant in the specimens with a greater groove depth. As shown in Figure 4(a,d), for a displacement of *δ* = 10 mm, the normalized power ratios *η̄* for groove depths of *h* = 0.0 and 1.1 mm are 0.96 and 0.88, respectively. The corresponding power losses are 0.04*η_o_* and 0.12*η_o_*, respectively. The sensitivity can be determined as 0.004 *η_o_*/mm and 0.012 *η_o_*/mm for groove depths of *h* = 0.0 and 1.1 mm, respectively. The results show that the sensitivity of the device can be improved significantly by increasing the groove depth. However, for a given groove depth, the variation of the power loss with the displacement is insensitive to the bend radius.

**Figure 5.**
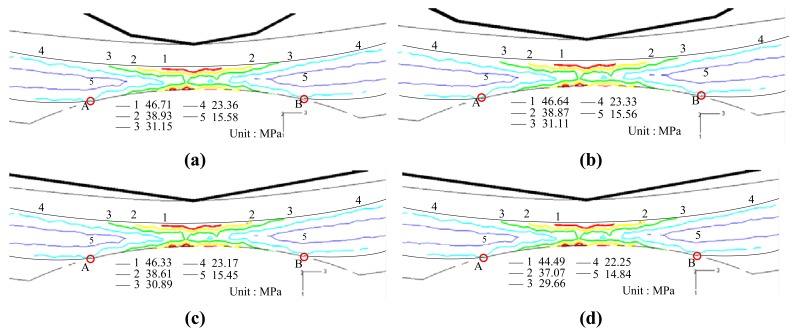
von-Mises stress contours near grooved region at *h* = 1.1 mm for various disc radii. (**a**) *R* = 5 mm. (**b**) *R* = 10 mm. (**c**) *R* = 15 mm. (**d**) *R* = 20 mm.

Figure 5 presents the FE simulation results for the von Mises stress distribution near the grooved region of the POF specimens deformed using various disc radii (note that the groove depth is 1.1 mm and the disc displacement is *δ* = 10 mm in every case). Comparing the four figures, it is seen that the disc radius has only a small effect on the von Mises stress distribution. From inspection, the maximum von Mises stress is found to be 46.71, 46.64, 46.33 and 44.49 MPa given disc radii of *R* = 5, 10, 15 and 20 mm, respectively.

As shown in Table 1, the yield stress of the core material (PMMA) is equal to 56 MPa. In other words, the FE results imply that the core material undergoes elastic deformation only at the maximum disc displacement of *δ* = 10 mm irrespective of the bend radius applied. In addition, Figure 5 shows that the length of the deformation region in the grooved POFs, *i.e.*, length $\bar{\text{AB}}$, is approximately the same in every case.

In [17], it was shown that a non-uniform stress distribution within POFs results in an inhomogeneous distribution of the refractive index and therefore prompts scattering losses. Since in the present study, there is no significant difference in the geometrical deformation or stress distribution in the various deformed POF specimens, the difference in the power loss induced by the discs of different radii is very small. In other words, as shown in Figure 4, the bend radius has no significant effect on the variation of the power loss with the bending displacement. In addition, since the deformation of the POFs remains within the elastic range, the normalized power ratio *η̄* is restored to a value of 1 once the disc load is released.

**Figure 6.**
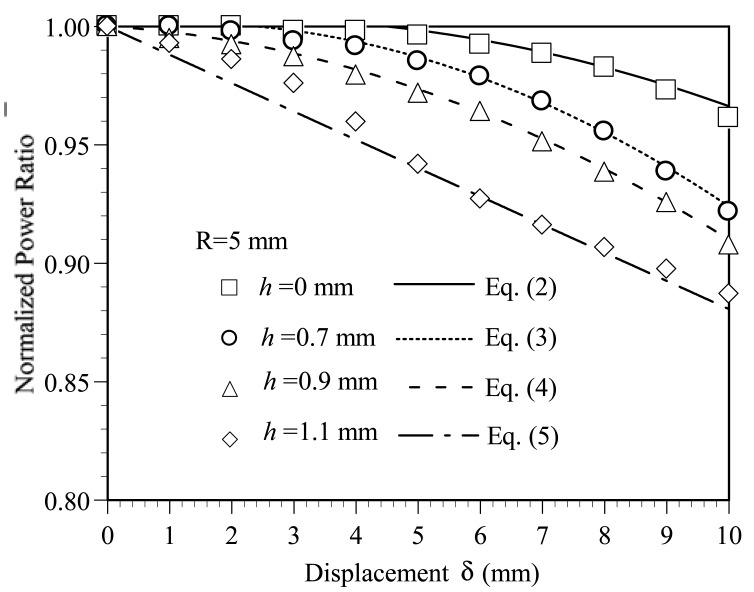
Variation of normalized power ratio *η̄* with displacement as function of groove depth *h*.

**Figure 7.**
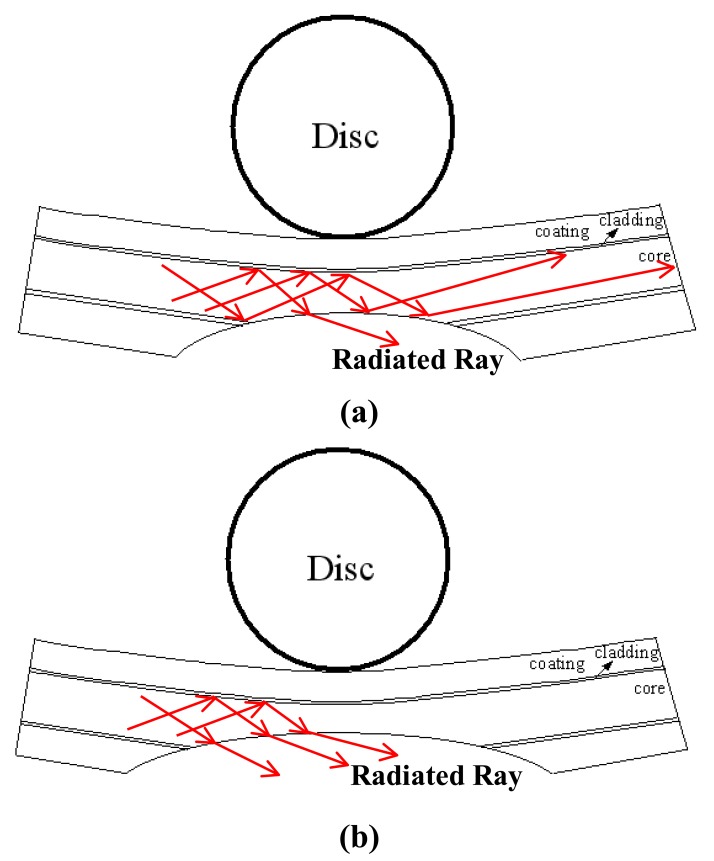
Ray paths in deformed POF specimens at various groove depths. (**a**) *h* = 0.9 mm. (**b**) *h* = 1.1 mm.

Figure 6 shows the variation of the normalized power ratio *η̄* with the bending displacement *δ* given various values of the groove depth *h*. Note that the symbols shown in Figure 6 represent the experimental results, while the dashed and solid lines represent the results obtained using the empirical model presented below. Note also that the results correspond to a disc radius of *R* = 5 mm in every case. It is seen that the normalized power ratio *η̄* decreases, *i.e.*, the power loss increases, as the bending displacement increases. Moreover, for a constant bending displacement, the power loss increases significantly with an increasing groove depth. From inspection, the normalized power ratio at the maximum displacement of *δ* = 10 mm is found to be 0.96, 0.92, 0.90 and 0.88 given groove depths of *h* = 0.0, 0.7, 0.9 and 1.1 mm, respectively. In other words, the power loss reaches a maximum value of 12% given a bending displacement of *δ* = 10 mm and a groove depth of *h* = 1.1 mm. Figure 7(a,b) illustrates the ray paths within the core of the deformed POF specimens with a groove depth of *h* = 0.9 and *h* = 1.1 mm, respectively. Note that the incident angles of the rays shown in Figure 7 are assumed to be 70°, *i.e.*, the critical angle of the undeformed POF. The reduction in the incident angle caused by grooving and the subsequent exposure of the core to the air causes some of the rays to be refracted through the core. As a result, a power loss is induced. From Figure 7(a,b), it can be observed that Figure 7(b) has a larger deformed groove depth. Thus, the number of rays refracted through the core/air boundary increases. This results in the increase of power loss.

Applying a nonlinear least squares fitting procedure to the experimental data shown in Figure 6, it is found that the normalized power ratio *η̄* for different values of the groove depth can be formulated in terms of the bending displacement *δ* as follows:

(2) $${\bar{\eta }}_{h=0}=1+2.6\times 10^{-3}\delta -6\times 10^{-4}\delta^{2}$$

(3) $${\bar{\eta }}_{h=0.7}=1+2.4\times 10^{-3}\delta -1\times 10^{-3}\delta^{2}$$

(4) $${\bar{\eta }}_{h=0.9}=1-1.6\times 10^{-3}\delta -7.4\times 10^{-4}\delta^{2}$$

(5) $${\bar{\eta }}_{h=1.1}=1-1.2\times 10^{-2}\delta +6.5\times 10^{-6}\delta^{2}$$

The predicted results obtained from Equations (2–5) for the variation of the normalized power ratio with the displacement are shown by the dashed and solid lines in Figure 6. It is evident that a good agreement exists between the two sets of results for all values of the groove depth. From inspection, the deviation between the two sets of results is found to be less than 2%. In other words, the empirical model presented in Equations (2–5) provides a reliable means of determining the displacement given a knowledge of the POF groove depth and power loss. As shown above, the bend radius has no significant effect on the variation of the power loss with the bending displacement. The empirical model shown in Equations (2–5) can be used for all values of the bending radius considered in this study. In general, Figure 6 shows that the normalized power ratio *η̄* decreases more rapidly with an increasing bending displacement given a greater value of the groove depth. This means when using a POF fiber subject to combined bending and elongation for displacement sensing purposes, the sensitivity of the device can be improved by increasing the groove depth.

## 5. Conclusions

This study has performed an experimental and numerical investigation into the power loss in grooved POFs subject to combined bending and elongation deformation. The investigations have considered groove depths ranging from 0∼1.1 mm and bending radii of 5, 10, 15 and 20 mm. The deformation-induced stress within the various POF specimens has been examined using an elastic-plastic FE model. The results have shown that the power loss increases significantly with an increasing bending displacement or groove depth, but is insensitive to the bending radius. The power loss reaches a value as high as 12% given a groove depth of *h* = 1.1 mm and a bending displacement of 10 mm. Based on the experimental results, an empirical model has been proposed for relating the power loss to the displacement given a knowledge of the groove depth. It has been shown that the value of the power loss predicted by the empirical model deviates by no more than 2% from the experimental value. Thus, the model provides a reliable means of determining the displacement given a knowledge of the power loss and groove depth, respectively. The grooved region of the sensor can be packed with a heat shrinkable tube to avoid the influence of humidity. However, the power loss may be affected by the material of the heat shrinkable tube. One of the possible applications of this study may be the displacement measurement within small confined areas, such as landslide detection and warning. Meanwhile, the possible indoor applications of this study may be the detection of cracks in concrete beams loaded in bending. In this application, the presence of cracks produces elongation on the POF and therefore attenuates the light transmission in it. The other application may be the detection of displacement in the structure of a building for earthquake damage evaluation. The durability and the influence of humidity on the POF specimen in long-term are important issues and will be taken into consideration in the future study.

## Figures and Tables

**Table 1.** Mechanical properties of POF materials [17,19,20].

**Property**	**Core (PMMA)**	**Cladding (PTFE)**	**Coating (LDPE)**
*E* (MPa)	3000	200	100
*σ_y_* (MPa)	56	11.3	9.8
*σ_ult_* (MPa)	72.4	55	17.15
*ν*	0.4	0.46	0.49
